# Genetic divergence of rubber tree estimated by multivariate techniques and microsatellite markers

**DOI:** 10.1590/S1415-47572010005000039

**Published:** 2010-06-01

**Authors:** Lígia Regina Lima Gouvêa, Luciana Benchimol Rubiano, Alisson Fernando Chioratto, Maria Imaculada Zucchi, Paulo de Souza Gonçalves

**Affiliations:** 1 Programa Seringueira, Instituto Agronômico, Campinas, SPBrazil; 2 Centro de Pesquisa e Desenvolvimento de Recursos Genéticos Vegetais, Instituto Agronômico, Campinas, SPBrazil

**Keywords:** genealogy, genetic diversity, *Hevea brasiliensis*, multivariate analysis, SSRs

## Abstract

Genetic diversity of 60 *Hevea* genotypes, consisting of Asiatic, Amazonian, African and IAC clones, and pertaining to the genetic breeding program of the Agronomic Institute (IAC), Brazil, was estimated. Analyses were based on phenotypic multivariate parameters and microsatellites. Five agronomic descriptors were employed in multivariate procedures, such as Standard Euclidian Distance, Tocher clustering and principal component analysis. Genetic variability among the genotypes was estimated with 68 selected polymorphic SSRs, by way of Modified Rogers Genetic Distance and UPGMA clustering. Structure software in a Bayesian approach was used in discriminating among groups. Genetic diversity was estimated through Nei's statistics. The genotypes were clustered into 12 groups according to the Tocher method, while the molecular analysis identified six groups. In the phenotypic and microsatellite analyses, the Amazonian and IAC genotypes were distributed in several groups, whereas the Asiatic were in only a few. Observed heterozygosity ranged from 0.05 to 0.96. Both high total diversity (H_T'_ = 0.58) and high gene differentiation (G _st'_ = 0.61) were observed, and indicated high genetic variation among the 60 genotypes, which may be useful for breeding programs. The analyzed agronomic parameters and SSRs markers were effective in assessing genetic diversity among *Hevea* genotypes, besides proving to be useful for characterizing genetic variability.

## Introduction

The *Hevea* genus belongs to the Euphorbiaceae family and comprises 11 species native to the Amazon region (Pires *et al.*, 2002). *Hevea brasiliensis* (Willd. ex Adr. de Juss.) Muell-Arg. is the only cultivated species and the main source of natural rubber.

Until about 1913, Brazil was the major producer of natural rubber, which was obtained from wild rubber trees growing in the rain forest of the Amazon basin. However, with the introduction of the Wickham material in 1876, Southeast Asia has gradually become the major producer of natural rubber accounting for more than 90% of the total production worldwide. There are approximately 7 to 8 million hectares of rubber plantations in the rubber areas of Asia and Africa. Genetic improvement through mass selection and modified recurrent selection has resulted in the production and release of elite clones, especially from Malaysia, over the past 60 years (Onokpise, 2004). Currently, *Hevea brasiliensis* is cultivated in several tropical countries, most of which have active plant-breeding programs (Sedgley and Attanayake, 1988).

In the past, there were limited numbers of *H. brasiliensis* clones suitable for use as parents in breeding programs, most of those available having already been selected according to phenotypic performance. They were crossed in many possible combinations, with posterior selection of the most promising families and progenies. Nowadays, an increased number of potential parents are available as a result of substantial breeding efforts and the exchange of clones among research institutions. Consequently, a wide range of crosses can now be attempted, this requiring additional resources for the effective exploitation and wise choice of parental clones.

Estimates of genetic divergence, through multivariate analysis of both agronomic characters and molecular markers, should provide valuable data for parent-choice in breeding programs. Multivariate analysis based on phenotypic data has been used to assess genetic diversity of rubber tree (Paiva, 1994; Omokhafe and Alika, 2003), as has also occurred with many other plant species, such as the assai palm (Oliveira *et al.*, 2007), coffee (Fonseca *et al.*, 2006) and bean (Chiorato *et al.*, 2007). More recently, molecular markers have proved to be useful in estimating genetic diversity in a wide range of species and populations. Among molecular markers, microsatellites or SSRs (Simple Sequence Repeats) have received special attention. These, besides being codominant and multi-allelic, are widely distributed throughout genomes, and thus can be highly polymorphic (Chin *et al.*, 1996). Of particular interest to geneticists and breeders, the SSR markers have been successfully used to infer about genetics, pedigree, phylogeny, and/or identity of various traits and/or germplasm accessions (McCouch *et al.*, 2001). SSR markers have been used to determine genetic diversity in several species, including maize (Laborda *et al.*, 2005), rice (Kwon *et al.*, 2002), common beans (Benchimol *et al.*, 2007) and rubber trees (Lekawipat *et al.*, 2003; Feng *et al.*, 2009).

The present study reports the suitability of *H. brasiliensis* microsatellite markers, developed from the GenBank database, for evaluation of genetic diversity in rubber tree clones. Furthermore, the estimates of molecular genetic divergence were compared with multivariate phenotypic analysis with the objective of exploring the feasibility of using SSRs for identifying superior crosses in breeding programs.

## Materials and Methods

Sixty *Hevea* genotypes (Table 1) from the Rubber Tree Program of the Agronomic Institute (IAC, Campinas, SP, Brazil) were chosen at advanced evaluation phases within genetic breeding programs. The selected genotypes consisted of Asiatic, African, Amazonian and IAC clones. Several of the Asiatic genotypes were derived from the Wickham collection originally introduced into Asia in 1876, and which are known as Wickham clones. The Amazonian clones were derived from selection and crossings carried out in Brazil by Ford and The North Agronomic Institute. They are the result of crossings among Amazonian and highly productive Asiatic genotypes, with the exception of the RO 45 clone, which was derived from a native plantation exploited for rubber extraction in the state of Rondônia (Brazil). The IAC clones resulted from controlled crossings and open pollinations performed in this research institute.

###  Phenotypical multivariate statistical analysis

Average values of five agronomical descriptors, each based on three replicates, were subjected to multivariate analysis. They comprised average of seven years of girth growth increment at juvenile immature phase before tapping, average of three years of girth growth increment in adult trees on tapping, average of three years of dry rubber yielding, virgin bark thickness in opened panel tapping; and the total number of latex vessel rings. These data were collected over a period of ten years. Measurements were taken as described by Gonçalves *et al.* (2006).

Multivariate procedures consisted of Standard Euclidian Distance (SED), Tocher Clustering and Principal Component Analysis (PCA). The contribution of each variable to genetic divergence was calculated by the criteria of Singh (1981). Statistical analyses were performed using the Genes software (Cruz, 2006).

###  SSR development and characterization

Total genomic DNA samples were extracted from powdery lyophilized leaf tissues using the 2% CTAB method (Hoisington *et al.*, 1994) with few modifications. A total of 470 reads from GenBank were evaluated in the development and characterization of *Hevea* microsatellites. Redundancies were identified using BLASTN software search utilities in GenBank (Altschul *et al.*, 1990). The SSR motifs in the sequences were identified, counted and localized by using SSRIT (Simple Sequence Repeat Identification Tool_ software. A total of 80 primer pairs (Table S1) were developed using Primer Select software from the Lasergene program (DNASTar, Inc.).

PCR amplifications were carried out in a 25 μL volume containing 100 ng of DNA, 1 U *Taq* DNA polymerase, 1.5 mM MgCl_2_, 200 μM of a total dNTP mixture and 0.8 μM of each forward and reverse primer. Each SSR was characterized on a gradient amplification profile, by varying the annealing temperature (*Ta*) at a difference of up to 10 °C. After an initial denaturing step of 1 min at 94 °C, the PCR amplification was performed in 30 cycles of 1 min at 94 °C, 1 min at the specific *Ta* and 1 min at 72 °C, followed by a final extension at 72 °C for 5 min and then kept at 15 °C. Alternatively, some SSRs could only be amplified by Touchdown. Amplification products were resolved on 6% (w/v) denaturing polyacrylamide gels and silver stained, according to Creste *et al.* (2001).

###  Polymorphism analysis, genetic distances and SSR clustering

Data on the presence (1) or absence (0) of SSR bands were transformed into genotypic data in order to identify loci and alleles. The Polymorphism Information Content (PIC) value for each locus was calculated using the PIC formula = 
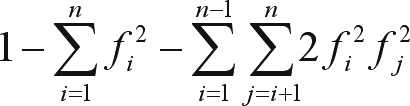
, in which *n* is the number of alleles; and *f*_*i*_ and *f*_*j*_ are the frequencies of the *i*^th^ and *j*^th^ alleles, respectively (Botstein *et al.* 1980).

Genetic distances were calculated by using Modified Rogers Genetic Distance (MRD) according to Goodman and Stuber (1983). A genetic distance matrix was estimated using TFPGA software. Cluster analyses were performed using UPGMA with the NTSYS-pc computer package version 2.02E. Clustering stability was tested by the Bootstrap procedure based on 10,000 re-sampling using the BooD program (Coelho, 2002). The cophenetic coefficients between the genetic distance matrix and the dendrogram derived matrix were performed using the NTSYS-pc computer package. The significance of cophenetic correlations was tested by applying Mantel correspondence analysis. Principal Coordinate Analysis (PCO, Gower 1966) was performed using MRD distance matrix. Genetic diversity among genotypes was estimated by way of Nei statistics using FSTAT Software.

**Figure 1:**
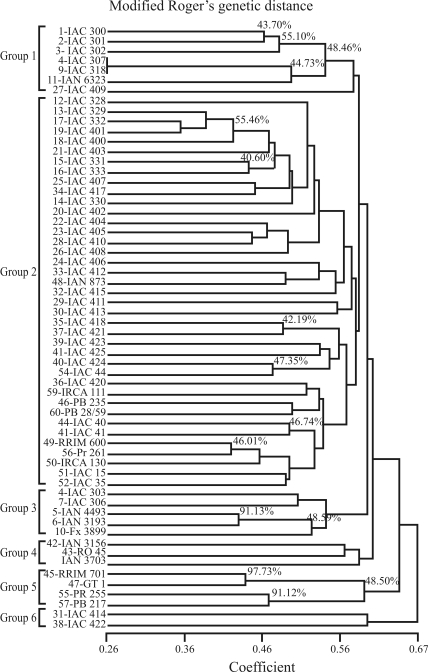
UPGMA cluster analysis of Modified Rogers Genetic Distances based on data from 68 SSRs, used in the evaluation of the 60 rubber-tree genotypes. Bootstrap node support, represented in percentages, shows clustering stability. Numbers (%) on the branches correspond to bootstrap values above 40% (10,000 replications). (Cophenetic value = 0.78).

The SED and MRD dissimilarity matrices were correlated using the Genes software (Cruz, 2006). Both the t- and Mantel tests were employed with 10,000 simulations to attribute significance values to the data. Intra- and inter-group correlations were performed using pair-wise genetic distances within and among groups, separated according to the group association pattern observed in the dendrogram (Figure 1).

The Bayesian approach of Pritchard *et al.* (2000) implemented by Structure software 2.2, was utilized alternatively to infer clustering. The number of clusters was defined from *K* = 3 to *K* = 20, and ten runs of each *K* were conducted using the admixture model and correlated allele frequencies, a 200,000 burn-in period and 500,000 MCMC. *Ad hoc* statistics was related to rate changes in the log probability of data according to the number of *K*s proposed by Evano *et al.* (2005), with Δ*K* being used as a predictor of the ideal number of clusters. In addition, the ideal number according to Pritchard and Wen (2004) was used as the criterion for defining the number of groups (k). The most trustworthy value was estimated based on the lowest negative number of Ln (the log-likelihood of the data) and the lowest standard deviation found during statistical analysis.

## Results

###  Phenotypic analysis

The 15 most divergent genotype pairs identified by the SED matrix are listed in Table 2. *Hevea benthamiana* was a common ancestor for seven of the 15. The most divergent genotype pair was IAC 318 - PB 235.

Through Tocher analysis, the 60 rubber-tree genotypes were clustered into 12 groups (Table 3). Among these, the eight Amazon genotypes (IAN 6323, IAN 3156, RO 45, IAN 4493, IAN 3193, IAN 873, IAN 3703 and Fx 3899) were distributed into five groups (I, II, VI, VII, VIII), and the 42 IAC genotypes into nine (I, III, IV, V, VI, VII, X, XI, XII). These results indicate high genetic diversity in the IAC and Amazon genotypes. Diversity in the eight Asiatic genotypes (RRIM 701, GT 1, PR 255, PB 217, RRIM 600, PR 261, PB 28/59 and PB 235) was low, with the majority, except for PB 235, PB 217 and RRIM 701, being allocated to group 1. The two African genotypes (IRCA 111 and IRCA 130) were clustered into one and the same group (IV). In Group 1, 56% of the genotypes proved to have either of the Indonesian clones, Tjir 1 or GT 711, in their ancestry. Notwithstanding, ancestry was not considered to be a suitable criterion for characterizing most of the groups.

PCA for phenotypic data accounted for 80.88% of the total variation in the first three principal components. The average dry rubber yield was the variable that contributed the most in the estimation of the genetic divergence among the 60 genotypes. The number of latex vessel rings was the least important variable, and so could be discarded.

###  Molecular analysis

Of the 80 characterized SSRs (Table S1) 68 were polymorphic and informative. In the SSR IAC-Hv34 genotype, two distinct bands were amplified and considered as two distinct loci (Hv34a, Hv34b). Thus, 69 polymorphic and informative SSR loci were identified and characterized. The electrophoretic profile obtained with SSR IAC-Hv72 can be observed in Figure S1. Polymorphic information content varied from 0.11 to 0.87, with an average of 0.57. The mean allele number per locus was 5.88, ranging from 2 to 13, the extremes being attributed to SSR IAC-Hv36 and SSR IAC-Hv20, respectively.

Six SSRs (IAC-Hv67, IAC-Hv68, IAC-Hv76, IAC-Hv44, IAC-Hv69 and IAC-Hv66) were efficient at amplifying *Hevea pauciflora*, and showed transferability in relation to *Hevea brasiliensis*, using the same PCR amplification procedure.

Observed heterozygosity (H_o_) based on Nei's estimates varied from 0.05 to 0.96, with an average of 0.45 (Table 4). Extreme H_o_ values were encountered in SSR IAC-HV09, IAC-HV66 and IAC-HV76. High total diversity (H_T'_ = 0.58) and high gene differentiation (G_st'_ = 0.61) were observed among all the 60 genotypes. SSR PCO accounted for 19.66% of the total variation in the first three axes. When selecting the most divergent pairwise distance in the Rogers modified genetic distance matrix (Table 5), IAC 414 appeared in eight of the pairwise distances. Dendrogram analyses (Figure 1) revealed six distinct groups. The eight Asiatic genotypes (RRIM 701, GT 1, PR 255, PB 217, RRIM 600, PR 261, PB 28/59 and PB 235) were distributed in only two groups (II, V). The African genotypes (IRCA 111 and IRCA 130) were clustered in group II. On the other hand, all the IAC genotypes were distributed among four groups (I, II, III and VI) while the eight evaluated Amazonian genotypes (IAN 873, IAN 6323, IAN 4493, IAN 3193, Fx 3899, IAN 3156, RO 45 and IAN 3709) were distributed in groups I, II, III and IV.

**Figure 2:**
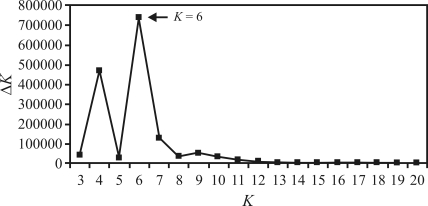
Representation of the number of ideal clusters identified by Structure software according to the methodology of Evano *et al.* (2005). The analysis was based on 68 SSRs utilized in the evaluation of the 60 rubber-tree genotypes.

Bootstrap analysis expressed high statistical node support for genotypes with shorter distances (Figure 2). The cophenetic correlation was r = 0.78 (p < 0.002). Groups were clearly distinguished, with several clusters being supported by high bootstrap values. Bootstrap analysis and cophenetic correlations indicated that SSR dendrogram clustering accurately depicted estimated genetic distances among rubber-tree genotypes. Group 1 contained all the genotypes derived from the AVROS clones. Group 2 comprised genotypes with either the GT 711 or RRIM 600 clone in their ascendancy. All the GT 711 derived genotypes were clustered in Group 2, and all the RRIM 600, but one (IAC 318), in Group 1. The IAC 400 genotypes were clustered in Group 2, except for two that were positioned in Group 6. The Amazonian genotypes IAN 4493, IAN 3193 and Fx 3899 were clustered in Group 3. Group 4 included the other three Amazonian clones IAN 3156, RO 45 and IAN 3703. The Wickham clones RRIM 701, GT1, PR 255 and PB 217 were gathered in Group 5. The IAC 414 and IAC 422 clones, placed in Group 6, were the only ones of the IAC 400 series outside Group 2.

**Figure 3:**
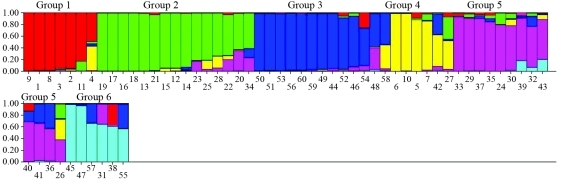
Distribution of the 60 rubber-tree genotypes in groups according to Structure analysis (k = 6), based in 68 SSRs used in the evaluation of the 60 rubber-tree genotypes. The individuals were represented by vertical bars, each color being associated to a different group. Genotype identification is referred to in Table 1.

A total of six groups were identified by Δ*K* as being the ideal number of groups, as previously proposed by Evano *et al.* (2005), and according to criteria indicated by Pritchard and Wen (2004). In an investigation of correspondence between the dendrogram and structure groups (Figure 3), group 1 of the dendrogram corresponded entirely to Structure group 1, and included genotypes derived from crosses of enhanced clones from the Rubber Research Institute of Malaysia (RRIM) and Algemene Verneiging Rubber planters Oostkust Sumatra of Indonesia (AVROS). Group 2 in the dendrogram corresponded to Structure groups 2, 3 and 5. Dendrogram group 3 corresponded to Structure group 4, and was characterized by Amazonian clones. Dendrogram group 5 and 6 corresponded to Structure group 6, and included four clones of the Wickham collection.

An interesting clustering aspect, as revealed by the structure program, was the distribution of the Amazonian and IAC genotypes into several groups, viz., I, III, IV, V and I, II, III, IV, V, VI, respectively, whereas the Asiatic genotypes (RRIM 701, GT 1, PR 255, PB 217, RRIM 600, PR 261, PB 28/59, PB 235) were distributed into only two groups (III, VI). This distribution pattern is in agreement with the data obtained through Tocher analysis of phenotypic data and the UPGMA dendrogram based on SSRs.

Matrix correlation between both kinds of genetic distances was significant by t-test and Mantel test (r = 0.13, p < 0.01). Genetic distances estimated from phenotypic and molecular traits were correlated. Pair-wise distances within and among groups were separated in the dendrogram according to the respective group association pattern (Figure 1). Significant values were found for intra-Group 2 correlations (r = 0.165, p < 0.01) and for inter-Group 1x3 correlation (r = 0.565, p < 0.01); inter-Group 1x5 (r = 0.547, p < 0.01) and inter-Group 1x6 (r = 0.620, p < 0.05).

## Discussion

###  Phenotypic analysis

Standard Euclidian Distances (SED) detected higher divergence between the clone IAC 318 and the Asiatic clone PB 235, the latter having been derived from a crossing between two Malayan clones. This dissimilarity possibly occurred through IAC 318 has the clone Fx 3899 as male parental, which is an interspecific hybrid of *H. benthamiana* x *H. brasiliensis* (Table 1). Among the 15 pairs of most divergent genotypes (Table 2), *H. benthamiana* appears as the ancestor in seven pairs. Hybridization may have several evolutionary consequences, these possibly including increased intra-specific genetic diversity (Rieseberg, 1997).

Total variance (80.8%), as explained by the three principal components of the phenotypic data, was expressive. However, it was less than that described by Paiva (1994), consisting of 94.76% and 97.49% data variance in the first three components with eight and seven descriptors, respectively. The number and nature of variables certainly have to be taken into consideration on comparing relative final variance.

###  Molecular analysis

Polymorphic information content was high for SSR loci, and indicated a substantial genetic information content in the clones analyzed with microsatellites. Microsatellite marker analysis is very efficient when examining genetic diversity (Laborda *et al.*, 2005; Saha *et al.*, 2005). PIC values for SSR loci were superior to those observed by Feng *et al.* (2009) when using EST-SSRs to analyze cultivated clones in rubber trees. Accordingly, PIC values ranged from 0 to 0.684 and averaged 0.383. As expected, EST-SSRs have been reported as being less polymorphic than genomic SSRs in crop plants due to DNA sequence conservation in transcribed regions (Scott *et al.*, 2000; Eujayl *et al.*, 2001). The mean allele number found in this study was 5.88, varying from 2 to 13. This result was similar to the previously reported 5.92, which varied from 3 to 10, in a set of cultivated genotypes (Lekawipat *et al.*, 2003), and higher than the average of 2.47 alleles observed by Feng *et al.* (2009), when using EST-SSRs.

Gene differentiation (G_ST_' = 0.61) was high, indicating 61% of total variation to be exploited among the available genotypes. A total of 52 SSRs presented high coefficients of genetic differentiation (G_ST_' > 0.50). This value was higher than that observed for other open pollinated species belonging to the same family as rubber trees, such as cassava (Fregene *et al.*, 2003; Lokko *et al.*, 2006). Observed heterozygosity was more variable than that described by Saha *et al.* (2005) in a cultivated rubber tree genotype when using four SSR markers, and therefore considered highly informative. This probably accounts for the lower variation observed.

Genomic transferability of the SSR *loci* between *H. brasiliensis* and *H. pauciflora* indicated that these SSRs could be useful for studies of synteny within the *Hevea* genus. Saha *et al.* (2005) also observed that SSRs specifically developed for *H. brasiliensis* efficiently amplified *H. benthamiana* and *H. spruceana*, thereby implying the high conservation of flanking microsatellite genomic regions. More recently, Feng *et al.* (2009) developed EST-SSRs for *H. brasiliensis*, and observed interspecies transferability by amplifying *H. spruceana*, *H. nitida*, *H. benthamiana* and *H. pauciflora*, and intergenus transferability in castor oil plants (*Ricinus communis* L.) and cassava (*Manihot utilissima*).

What makes the Bayesian approach interesting in the study of population genetic structures is the facility in detection without the need for prior information on individual origin (Pritchard *et al.*, 2000). When considering all the individuals and clustering in Structure at *K = 6*, the arrangement was such as to correspond to dendrogram clustering. The coherence in genotype clusters indicated a non-random distribution of alleles and their frequencies. In fact, clones in most groups were gathered according to ascendancy as previously described. The lack of consistency in various dendrogram clusters could be associated to low bootstrap node support (with 10,000 re-samplings) in some of the major groups. Low bootstrap nodes could be associated with the lack of genetic structure, and the incapacity of a clearing clustering tendency supported by high cophenetic values.

###  Phenotypic and Molecular analysis

On comparing phenotypic and molecular clustering patterns (Table 3; Figure 1), molecular markers have proved to be very efficient in group characterization by genealogy. In rubber tree studies using molecular markers (Varghese *et al.*, 1997; Feng *et al.*, 2009), genealogy has also been used as an aid in group characterization, although not always with satisfactory results. In this work, illegitimate genotypes derived from open pollination with the same female parental, whereas those legitimate were the result of controlled pollination, with both common parental belonging to separate groups. According to Varghese *et al.* (1997) *Hevea* being a predominantly cross-pollinated tree species, has F1 hybrids fixed vegetatively while clones are highly heterozygous. As a result of segregation and independent assortment in these clones, the proportion of marker alleles in the F1 hybrid from each parent can vary considerably. Thus, in highly heterozygous species with a common ancestry, pedigree information may not always reveal the exact nature of genetic relationships.

In phenotypic and microsatellite analyses, both the Amazonian and IAC genotypes were clustered into several groups, thereby indicating high genetic diversity among these genotypes. On the other hand, Asiatic genotypes were distributed in only a few groups, thus indicating low diversity, with, most certainly, a narrow genetic base (Besse *et al.*, 1994; Varghese *et al.*, 1997). Molecular analysis indicated the close relationship between African and Asiatic genotypes, thereby confirming their Asiatic genetic base (Besse *et al.*, 1994). The data confirmed a narrow genetic base for the Asiatic and African genotypes and high genetic variability for the Amazonian genotypes. This higher genetic variability in wild Amazonian genotypes was expected, although it is not always associated to desirable agronomic breeding characters. Clément-Demange *et al.* (2001) reported that Amazonian wild genotypes do not always contribute desirable traits to rubber tree genetic breeding. Even though, Amazonian wild genotypes were introduced into rubber tree breeding by crossing, so as to widen the genetic basis of Asiatic clones. The Amazonian genotypes evaluated in this study arose from prior breeding and selection procedures, thereby possibly constituting an interesting genetic background to be exploited in rubber tree breeding programs. Indeed, these clones showed the highest genetic diversity when compared to the other improved clones analyzed, and appear to be attractive for rubber breeding.

The most divergent genotypes identified in SED analysis (Table 2) differed from those identified through MRD analysis (Table 5). *H. benthamiana* was an ancestor in seven of the 15 most dissimilar SED genotype pairs. Moreover, the rubber tree clone IAC 414 was the most dissimilar MRD genotype, being involved in eight of the 15 largest distances registered. It should be noticed that IAC 414 has the Amazonian Fx 25 clone as an ancestor. Although Asiatic genotypes ancestry was predominant in the majority of the evaluated rubber tree clones, higher genetic divergence was observed in those genotypes derived from inter-specific crosses performed in the past, as well as those having the Fx 25 clone as ancestor. The data indicated the strong contribution of these two genotypes to genetic divergence as described in this study. Further analysis should be undertaken to confirm these findings.

Although both phenotypic and molecular analysis revealed differences in genotype clustering, they shared several common aspects, such as high diversity between Amazonian and IAC genotypes and low among Asiatic. Most likely, the difference in genotype clustering was due to agronomic descriptors being associated to gene expression and may suffer environmental influence modulating the phenotype. In contrast, molecular markers, including microsatellites, are mostly neutral and consist of either expressed DNA sequences, or non-expressed genomic regions such as introns or regulatory sites. In agreement with Moser and Lee (1994), as a result of the complex nature of the relationship between phenotypic and genotypic variation, genotypes that are phenotypically different may vary at only a few loci, and those very similar in appearance or performance may be quite dissimilar genetically. In addition and as reported by Grivet and Noyer (2003), the relationship between neutral polymorphism revealed by markers and the polymorphism of useful morpho-agronomic characters is not clear. Markers revealed sequence similarity between individuals in a sample of a locus. Morpho-agronomic characters measure resemblances between individuals based on variables whose level of expression depends on the number of potentially epistatic genes.

Despite being significant, matrix correlation for all genetic distances of different marker systems exhibited poor association, which was also observed by Roldán-Ruiz *et al.* (2001). Taking into account the molecular dendrogram clustering and correlated genotypes, significant associations were detected between intergroup genetic distances (phenotypic and SSR-based GDs) in Groups 1x3; 1x5 and 1x6. Currently, associations between molecular and phenotypic data tend to be stronger in crosses between genotypes of similar pedigrees (Smith *et al.*, 1990). Certainly, correlations between phenotypic-based and molecular-based distances will be improved as additional probes or marker loci are employed in analysis (Moser and Lee, 1994).

The genotypes analyzed in the present work had been previously selected and evaluated in breeding programs, and had demonstrated good performance. Thus, prior screening of the most divergent genotype pairs identified through both methods is suggested for evaluation of the relative agronomic performance of their hybrids. In a traditional breeding program, thousands of crosses are normally performed and evaluated in experimental designs. According to the data described in this work, SSR-based genetic distances could be useful in selecting superior crosses between rubber tree clones derived from a population with a broad genetic base. Hence, the application of SSR markers in rubber-tree breeding could be instrumental in reducing the number of single-cross hybrids to be evaluated. SSRs are easily assayed by the Polymerase Chain Reaction, and have proved to constitute a potent tool for characterizing genetic diversity. Furthermore, in perennial plant species, such as rubber trees, this molecular marker technology has the additional advantage of shortening breeding time by allowing for the screening of seedlings and juvenile plants.

## Supplementary Material

The Following online material is available for this article:

Table S1 Sequence of primers and gene annotations.

Figure S1 SSR IAC-Hv72 electrophoretic profile of rubber tree genotypes on 6% polyacrylamide gel.

This material is available as part of the online article from http://www.scielo.br/gmb.

## Figures and Tables

**Table 1:** Sixty rubber tree genotypes selected in the breeding program of the Instituto Agronômico (IAC) and their respective genealogy.

Nº	Clone	Genealogy
1	IAC 300	RRIM 605(Tjir 1 x PB 49) x AVROS 353(AVROS 164 x AVROS 160)
2	IAC 301	RRIM 501(Pil A 44 x Lun N) x AVROS 1518(AVROS 214 x AVROS 317)
3	IAC 302	RRIM 501 (Pil A 44 x Lun N) x AVROS 353(AVROS 164 x AVROS 160)
4	IAC 303	RRIM 511(Pil A 44 x Pil B 16) x AVROS 1518 (AVROS 214 x AVROS 256)
5	IAN 4493	Fx 4421 (F 4573 x PB 86) x Tjir 1
6	IAN 3193	Fx 516(F 4542^(1)^ x AVROS 363) x PB 86
7	IAC 306	AVROS 49 x RRIM 509(Pil A 44 x Lun)
8	IAC 307	AVROS 1328 (AVROS 214 x AVROS 317) x PR 107
9	IAC 318	RRIM 600 (Tjir 1 x PB 49) x Fx 3899 (F 4542^(1)^ x AVROS 363)
10	Fx 3899	F 4542^(1)^ x AVROS 363
11	IAN 6323	Tjir 1 x Fx 3810 (F 4542^(1)^x AVROS 363)
12	IAC 328	RRIM 600 (Tjir 1 x PB 86) x PR 107
13	IAC 329	GT 711 x Tjir 16
14	IAC 330	RRIM 600(Tjir 1 x PB 86) x GT 711
15	IAC 331	RRIM 600(Tjir 1 x PB 86) x AVROS 1328 (AVROS 214 x AVROS 37)
16	IAC 333	C 228 x Tjir 16
17	IAC 332	GT 711 x RRIM 600 (Tjir 1 x PB 86)
18	IAC 400	GT 711 x RRIM 600 (Tjir 1 x PB 86).
19	IAC 401	RRIM 600 (Tjir 1 x PB 86) ill.
20	IAC 402	GT 711 ill
21	IAC 403	GT 711 ill.
22	IAC 404	PB 5/63 x AVROS 636
23	IAC 405	Tjir 1 x RRIM 323.
24	IAC 406	IAN 873 (PB 86 x FA 1717) x RRIM 600 (Tjir 1 x PB 86)
25	IAC 407	RRIM 600 (Tjir 1 x PB 86) ill.
26	IAC 408	RRIM 513 (Pil B 16 x Pil A 44) ill.
27	IAC 409	Fx 2784 (F 4542^(1)^ x AVROS 363)ill.
28	IAC 410	PB 86 x PB 235.
29	IAC 411	GT 711 ill.
30	IAC 413	IAN 873 (PB 86 x FA 1717) ill.
31	IAC 414	IAC 126 (Fx 25 (F 351 x AVROS 49) x Tjir 1) ill.
32	IAC 415	AVROS 363 ill.
33	IAC 412	IAN 873 (PB 86 x FA 1717) x GT 711
34	IAC 417	RRIM 600 (Tjir 1 x PB 86) ill.
35	IAC 418	RRIM 600 (Tjir 1 x PB 86) ill.
36	IAC 420	IAN 873 (PB 86 x FA 1717) ill.
37	IAC 421	IAC 157 [Fx 505(F 4542^(1)^ x AVROS 363) x Fx 25 (F351 x AVROS 49) ill.
38	IAC 422	RRIM 513 (Pil B 16 x Pil A 44) ill.
39	IAC 423	IAC 90 [RRIM 507(Pil B 84 x Pil A 44) x Fx 25(F 351 x AVROS 49) ill.
40	IAC 424	RRIM 600 (Tjir 1 x PB 86) ill.
41	IAC 425	RRIM 600 (Tjir 1 x PB 86) ill.
42	IAN 3156	Fx 516 (F 4542^(1)^ x AVROS 363) x PB 86
43	RO 45	Primary clone
44	IAC 40	RRIM 608 (AVROS 33 x Tjir 1) x AVROS 1279 (AVROS 156 x AVROS 374)
45	RRIM 701	44/553 x RRIM 501 (Pil A 44 x Lun N)
46	PB 235	PB 5/51 x PB S/78
47	GT 1	Primary clone
48	IAN 873	PB 86 x FA 1717
49	RRIM 600	Tjir 1 x PB 86
50	IRCA 130	PB 5/51 X IR 22
51	IAC 15	RRIM 504 (Pil A 44 x Lun N) x RRIM 600 (Tjir 1 x PB 86)
52	IAC 35	Fx 25 (F 351 x AVROS 49) x RRIM 600 (Tjir 1 x PB 49)
53	IAC 41	RRIM 608 (AVROS 33 x Tjir1) x AVROS 1279 (AVROS 256 x AVROS 374)
54	IAC 44	IAN 2325[PB 86 x Fx 3933 (F4542^(1)^ x AVROS 363)] x AVROS 1328(AVROS 214 x AVROS 3170]
55	PR 255	Tjir 1 X PR 107
56	PR 261	Tjir 1 X PR 107
57	PB 217	PB 5/51 X PB 69
58	IAN 3703	Fx 4371 [Fx 3472(F 4542^(1)^ x PB 86) x PB 86] x PB 86
59	IRCA 111	PB 5/51 x RRIM 600 (Tjir 1 x PB 49)
60	PB 28/59	PBIG seedling

^(1)^ Primary clone of *Hevea benthamiana.* Amazonian clones (F = Ford, FA = Ford Acre, Fx = Ford crossbred, IAN = Instituto Agronômico do Norte); Clones from the State of Sao Paulo (IAC = Instituto Agronômico); Asiatic clones (AVROS = Algemene Verneiging Rubber planters Oostkust Sumatra, Indonesia; GT = Godang Tapen, Indonesia; PB = Prang Besar, Malaysia; PR = Proefstation voor rubber, Indonesia; Pil = Pilmoor, Malaysia; RRIM = Rubber Research Institute of Malaysia, Malaysia; Tjir = Tjirandji, Indonesia); African clones (IRCA = Institute de Recherches sur le Caoutchouc, Ivory Coast).

**Table 2:** Fifteen pairs of the most divergent genotypes according to Standard Euclidian Distance (SED), estimated for 60 rubber-tree genotypes and considering five agronomic descriptors.

Order	SED	Genotype pairs
1°	6.41	IAC 318 - PB 235
2°	6.16	IAC 40 - RRIM 701
3°	6.11	IAC 318 - IRCA 130
4°	6.10	IAC 318 - IAC 401
5°	6.10	IAC 318 - IAC 400
6°	6.09	IAN 3156 - PB 217
7°	6.08	IAC 306 - IRCA 130
8°	6.06	IAN 3156 - RRIM 701
9°	6.05	IAC 306 - IAC 406
10°	5.99	IAC 306 - IAC 401
11°	5.98	IAC 40 - PB 217
12°	5.97	IAC 414 - PB 235
13°	5.85	IAC 306 - IAC 400
14°	5.80	IAC 414 - IAN 3156
15°	5.76	IAC 331- IAC 401

Clones from the State of Sao Paulo (IAC = Instituto Agronômico); Asiatic clones (PB = Prang Besar, Malaysia; RRIM = Rubber Research Institute of Malaysia, Malaysia), Amazonian clones (IAN = Instituto Agronômico do Norte); Clones from the State of Sao Paulo (IAC = Instituto Agronômico), African clones (IRCA = Institute de Recherches sur le Caoutchouc, Ivory Coast).

**Table 3:** Clustering of 60 rubber-tree genotypes by the Tocher method and based on dissimilarity estimated by Standard Euclidian Distance (SED), using five agronomic descriptors.

Groups	Genotypes
I	IAC 302, Fx 3899, IAN 6323, IAC 328, IAC 329, IAC 330, IAC 333, IAC 402, IAC 403, IAC 404, IAC 407, IAC 408, IAC 409, IAC 411, IAC 415, IAC 412, IAC 417, IAC 421, IAC 422, IAC 424, IAC 425, GT 1, IAN 873, RRIM 600, IAC 15, IAC 35, IAC 41, IAC 44, PR 255, PR 261, IAN 3703, PB 28/59
II	IAN 3193, RRIM 701, PB 217
III	IAC 332, IAC 413, IAC 414, IAC 418
IV	IAC 400, IAC 401, IRCA 130, IRCA 111
V	IAC 405, IAC 406, IAC 410, IAC 420
VI	IAC 300, IAC 301, IAC 303, IAN 4493, IAC 306, IAC 307
VII	RO 45, IAC 40
VIII	IAN 3156
IX	PB 235
X	IAC 423
XI	IAC 318
XII	IAC 331

Amazonian clones (Fx = Ford crossbred, IAN = Instituto Agronômico do Norte); Clones from the State of Sao Paulo (IAC = Instituto Agronômico de Campinas); Asiatic clones (AVROS = Algemene Verneiging Rubber planters Oostkust Sumatra, Indonesia; GT = Godang Tapen, Indonesia; PB = Prang Besar, Malaysia; PR = Proefstation voor rubber, Indonesia; RRIM = Rubber Research Institute of Malaysia, Malaysia); African clones (IRCA = Institute de Recherches sur le Caoutchouc, Ivory Coast).

**Table 4:** Gene diversity analysis of 60 rubber tree genotypes of the breeding program of the Instituto Agronômico (IAC) estimated by SSRs.

SSR	Ho	Ht'	Gst'		SSR	Ho	Ht'	Gst'
IAC-HV09	0.05	0.42	0.94		IAC-HV45	0.20	0.18	0.45
IAC-HV10	0.30	0.88	0.83		IAC-HV46	0.55	0.56	0.51
IAC-HV28	0.27	0.58	0.77		IAC-HV49	0.36	0.55	0.67
IAC-HV13	0.52	0.45	0.42		IAC-HV51	0.73	0.70	0.48
IAC-HV17	0.40	0.77	0.74		IAC-HV61	0.68	0.81	0.58
IAC-HV24	0.33	0.68	0.76		IAC-HV65	0.12	0.11	0.47
IAC-HV08	0.23	0.41	0.72		IAC-HV62	0.39	0.59	0.67
IAC-HV03	0.14	0.55	0.87		IAC-HV66	0.05	0.46	0.94
IAC-HV06	0.47	0.76	0.69		IAC-HV58	0.57	0.52	0.45
IAC-HV04	0.55	0.73	0.62		IAC-HV56	0.06	0.36	0.92
IAC-HV05	0.46	0.75	0.70		IAC-HV69	0.90	0.79	0.43
IAC-HV02	0.53	0.78	0.66		IAC-HV55	0.43	0.66	0.68
IAC-HV11	0.43	0.64	0.66		IAC-HV44	0.65	0.65	0.50
IAC-HV07	0.77	0.83	0.54		IAC-HV76	0.96	0.82	0.41
IAC-HV27	0.47	0.52	0.55		IAC-HV75	0.41	0.70	0.71
IAC-HV20	0.65	0.84	0.61		IAC-HV78	0.60	0.50	0.40
IAC-HV15	0.62	0.80	0.62		IAC-HV63	0.32	0.57	0.72
IAC-HV12	0.27	0.29	0.53		IAC-HV68	0.17	0.34	0.76
IAC-HV01	0.42	0.61	0.66		IAC-HV67	0.32	0.49	0.68
IAC-HV16	0.70	0.72	0.51		IAC-HV79	0.33	0.34	0.52
IAC-HV23	0.77	0.60	0.36		IAC-HV70	0.21	0.57	0.82
IAC-HV25	0.55	0.65	0.58		IAC-HV50	0.33	0.34	0.52
IAC-HV29	0.25	0.25	0.48		IAC-HV57	0.42	0.40	0.48
IAC-HV22	0.82	0.81	0.49		IAC-HV80	0.64	0.62	0.48
IAC-HV30	0.87	0.84	0.49		IAC-HV53	0.88	0.59	0.26
IAC-HV18	0.72	0.72	0.51		IAC-HV74	0.82	0.85	0.52
IAC-HV14	0.68	0.75	0.55		IAC-HV75	0.58	0.60	0.52
IAC-HV31	0.48	0.73	0.67		IAC-HV52	0.29	0.39	0.63
IAC-HV32	0.10	0.13	0.60		IAC-HV73	0.60	0.84	0.64
IAC-HV40	0.36	0.51	0.65					
IAC-HV33	0.67	0.79	0.58					
IAC-HV35	0.28	0.67	0.79					
IAC-HV42	0.38	0.53	0.64					
IAC-HV47	0.35	0.49	0.65					
IAC-HV34a	0.90	0.59	0.24					
IAC-HV34b	0.15	0.14	0.47					
IAC-HV49	0.48	0.65	0.63					
IAC-HV38	0.07	0.16	0.79					
IAC-HV60	0.17	0.56	0.85					
Overall	0.45	0.58	0.61					

Ho: observed heterozygosity. Ht': total heterozygosity. Gst': co-efficient of gene differentiation.

**Table 5:** Fifteen pairs of the most divergent rubber-tree genotypes according to Modified Rogers Genetic Distance (MRD) estimated among 60 rubber-tree genotypes evaluated with 68 SSRs.

Order	MRD	Genotype Pairs
1^st^	0.74	IAC 328 - PB 217
2^nd^	0.73	IAC 414 - IAC 41
3^rd^	0.73	IAC 414 - PB 217
4^th^	0.72	IAC 418 - RRIM 701
5^th^	0.72	IAC 330 - RRIM 701
6^th^	0.72	Fx 3899 - PB 217
7^th^	0.72	IAC 328 - IAC 414
8^th^	0.71	Fx 3899 - PR 255
9^th^	0.71	IAC 408 - IAC 414
10^th^	0.71	IAC 422 - IRCA 130
11^th^	0.71	IAC 401 - IAC 414
12^th^	0.70	IAC 333 - RRIM 701
13^th^	0.70	IAC 331 - IAC 414
14^th^	0.70	IAN 4493 - IAC 414
15^th^	0.70	IAC 414 - PR 261

Clones from the State of São Paulo (IAC = Instituto Agronômico de Campinas); Asiatic clones (PB = Prang Besar, Malaysia; PR = Proefstation voor rubber, Indonesia; RRIM = Rubber Research Institute of Malaysia, Malaysia); Amazonian clones (Fx = Ford crossbred; IAN = Instituto Agronômico do Norte); African clones (IRCA = Institute de Recherches sur le Caoutchouc, Ivory Coast).
