# Supplementary material for: Genetic divergence of rubber tree estimated by multivariate techniques and microsatellite markers
Source: Genet Mol Biol. 2010 Jun 1;33(2):308–18. doi: 10.1590/S1415-47572010005000039 (PMC3036869; doi:10.1590/S1415-47572010005000039)
Supplement: Table S1 — Sequence of primers and gene annotations. [file gmb-33-2-308-suppl1.pdf]

Table S1 - Sequence of primers and gene annotations.

Nº	Sequence ID	SSR	Motivos	Forward Primer (5'-3')	Reverse Primer (5'-3')	Ta (oC) [1]	No alelos	PIC[2]	Produto min-max
1	AF221702	IAC-Hv01	(ga)19	GAAGAAAAACAGAAACACATAA	CTACACCCCAGCAAAGAAT	62.0	6	0.604	188-210
2	AF383944	IAC-Hv02	(gt)19	CAGTAGAAAGGGGAATC	AACACTGAAAACAACAATG	55.0	11	0.773	130-170
3	AF486730	IAC-Hv03	(ga)21	AGTTACAACAATCATCATCTG	ATAAAGTCTTGGGAAAACAT	58.0	5	0.538	300-306
4	AY439312	IAC-Hv04	(ag)20	AAACCCATGCAAACGAAAGA	TTAAAACGGCAGTGGAAAGAAG	60.0	6	0.723	136-146
5	AY486731	IAC-Hv05	(ct)16	GTATCCCGAGTCGTCTTCAT	TATAAACTAAAATTGCCCTCAG	62.0	6	0.742	268-300
6	AY486741	IAC-Hv06	(ct)19	GGACTAAAACGATCTAATGCTA	AAGAGTCTGTGATGGAGTGAAG	58.0	9	0.748	234-270
7	AY486744	IAC-Hv07	(ga)16	CAGCCTACAGCAGATTTGAA	GGCTGCCATTTGTAGTTTTA	60.9	10	0.820	248-280
8	AY486747	IAC-Hv08	(ga)18	CCGAAGAAGGGTGTAAT	ATCAACTAGTGCCAAGAAG	TD	5	0.406	260-270
9	AY486749	IAC-Hv09	(ga)17	GGAAAAACAGCCCATTACTTG	CTGCGGCTCATAGGAAAAC	60.0	4	0.412	222-240
10	AY486753	IAC-Hv10	(ct)21	TATCAAAATCAGCAGCATCTAC	TCTCCAGTTGTTATCCTCTTCT	60.0	9	0.867	154-184
11	AY486754	IAC-Hv11	(ca)20	CTCGTCTTCTAACTCCGCTCTA	TATCGATATTGTGGTGCTTGAA	60.0	6	0.637	230-270
12	AY486769	IAC-Hv12	(ct)15(ca)13	TGCATGCATGAAGTGTCTAA	AAAAACCTTGAGGATGTAATG	63.0	5	0.287	134-142
13	AY486776	IAC-Hv13	(ca)15	CATGCAATTCACTACAAAACAA	GAAGCAGAACGACCATCAA	60.0	4	0.444	160-268
14	AY486777	IAC-Hv14	(gt)2gc(gt)5(gc)6(gt)7	CTCAAATCATATGGTGGAAGTG	ATGATGTTAAAAGGGGGAAGTT	63.0	8	0.745	134-160
15	AY486836	IAC-Hv15	(ga)25	ACTCGCTGACCATAAAAAGAAG	TATTGGGATCCTATTGAAAAGA	60.0	12	0.792	200-228
16	AY486838	IAC-Hv16	(ga)19	ACAACACGATTACCAAGGAG	AAAAAGGAGACAGGCACATA	60.0	6	0.714	220-232
17	AY486841	IAC-Hv17	(ct)15	TTACTTTATGCAACCTGATGAA	GTGGCAACTGAAAACTGAAG	60.0	6	0.764	136-146
18	AY486842	IAC-Hv18	(ga)16	TGCAGTCACATCCACAA	GGTCTTACTACATCATCAAAAT	60.0	7	0.718	134-150
19	AY486843	IAC-Hv19	(ga)24	TCTTTCCAACCTTCATCACAG	AGGATGGGAAGTAAGCTAGAAA	Not amplified			
20	AY486845	IAC-Hv20	(ct)18	GGGACATGGGGCTTATCTC	AGTGGCTTCATCTTCAAAACC	60.0	13	0.834	168-226
21	AY486850	IAC-Hv21	(ct)20	ATTAACCATTTCAACCAT	AATCCTTTCTTTATCTCC	TD			Acima 330pb
22	AY486854	IAC-Hv22	(ct)20	ACTTTATCTGGTTTTCGTATCA	GCCATGCATGTTGTGTTG	60.3	10	0.798	134-152
23	AY486856	IAC-Hv23	(ct)19	ATGGAGGAATAAAAGGGTCTA	GGGATGATTGTTTGAAGGATT	65.0	5	0.565	246-254
24	AY486857	IAC-Hv24	(ca)10(ga)18	AAAAGTTCGCCGTAAGATGA	TGAGGCCAAGAACAATAGAAT	60.0	6	0.671	260-270
25	AY486858	IAC-Hv25	(ga)25	GATGGAGAGCTGGTAAGAAGAA	ATCGAGGGAACTAATCAAACAA	61.8	6	0.648	178-188
26	AY486862	IAC-Hv26	(ga)4 caga(ca)12(ga)14	AACTGCGGTACGTGATTTTC	TTTGGAGGTTTAGGATTTGATT	Not amplified			
27	AY486870	IAC-Hv27	(ga)29	TGGGGACAAAGTGAGGTT	CAAAAGAAAAGAAAAAGAAATC	56.0	6	0.515	142-156
28	AY486874	IAC-Hv28	(ga)19	TGTGCTTTTGTAATAGGGAATC	TATGGTTTGAGCTGTGTTGTG	55.0	6	0.570	124-134
29	AY486876	IAC-Hv29	(ct)23	GGTGCTCCAGGTAAAGTG	GATAGTCGCTCATAGAAAACA	60.0	4	0.244	146-152
30	AY486877	IAC-Hv30	(ct)21	ATAGAATACCTGAACAATAAC	AGGGAACATCTAAATAAAT	54.0	8	0.838	148-160
31	AY486878	IAC-Hv31	(ct)21	CATATGCTTTGGTCTGTTCTCC	TCCAATGCCTGCCTCTTCTA	65.0	8	0.722	200-224
32	AY486884	IAC-Hv32	(ct)17	TTCACTCTTCTCGCATCAAAC	ACAAAACAACCGAACACAAACA	65.0	2	0.124	190-198
33	AY486889	IAC-Hv33	(ca)18	TATGGCTATGTTCTGCTATGC	ATGATTTTGGTTACCTGGAAGA	64.8	9	0.779	134-158
34	AY486892	IAC-Hv34	(ga)26	ATGCAAAATCACAGAAAATGGA	GGGCAGGGAAAGAGATGTCA	64.8	3	0.589	124-128
							3	0.141	218-234
35	AY486900	IAC-Hv35	gtga(gt)3ct(gt)8	ACGACCAGCTTCAGTTTA	CATGTGGTGCAAGTTTCT	60.5	3	0.657	148-152
36	AY962210	IAC-Hv36	(ct)1c(ct)8cg(ct)3	CAAAAGCCAATTAATACACATA	GTCACACCATCTACCAAATAAG	61.0	2	0.339	240-242
37	AY962212	IAC-Hv37	(ct)6(ca)14(ta)4	GTTAATTATACGCTGTGC	TCCAAGAGTCGCTAAAT	Not amplified			
38	AY962216	IAC-Hv38	(ag)4ca(ag)7	ATCCTCAATTCTTTCTTCA	AGTCTTCTTCTCTACCTCTC	TD	3	0.156	212-218
39	AY962217	IAC-Hv39	(ac)18	TAGGGACTTTTCTGGCTTTTTA	TGGATGTACCTGAGATTTTGAG	Not amplified			
40	AY962222	IAC-Hv40	(ag)15	GCTCGAGCCCATCTACT	ACTCCCTTCTCTATTCTCACTC	65.0	6	0.508	152-170
41	AY962227	IAC-Hv41	(ac)6ag(at)2(ac)22ag(at)27	GGGACGCATGTTGTGTATT	AAAAATGGGATGAGTTAGAGAT	Not amplified			
42	AY962229	IAC-Hv42	(ag)10atg(ag)3	CAGGGAGGCAGTGAGCA	AATTCCCTAGTTCTTTGTTGAC	65.0	4	0.520	148-156
43	AY962229_b	IAC Hv42b	(ag)10atg(ag)3	CAGGGAGGCAGTGAGCA	AATTCCCTAGTTCTTTGTTGAC				
44	AY962232	IAC-Hv44	(ga)11(gt)11	CTGGCGAGCTGGGAAGG	CGGCTGAATCGGACTGG	59.3	4	0.648	144-150
45	AY962235_A	IAC-Hv45	(aaat)5	ATCAGCACATTAACAACACA	TTATTTCTCTTTTCCTTTACTG	60.5	2	0.183	214-218
46	AY962235_B	IAC-Hv46	(ga)13	ATAGGAACAACAGCAACAGTAA	TGCTTTTCTTCGCTCTTCTT	65.0	8	0.558	168-182
47	AY962190	IAC-Hv47	(ga)7aa(ga)5	CGCTAGCACACTCAAGAAAACA	CCAACGGCTGAAATAGAACAAT	59.5	5	0.487	152-164
48	AF221697	IAC-Hv48	(ga)8 aa(ga)6	AGTTCACCGTGCTTATTTCA	AAGGCATTGTTCTGGGATTT	65.0			Acima 330 pb
49	AF383942	IAC-Hv49	(gt)18(tg)2	TGCAGCAGTTACATCACCAA	AGGGCCAGAGTCAAAAAGAGT	66.0	5	0.642	222-250
50	AY486791	IAC-Hv50	(gt)19	GCATAATAGTGACAGAAAGAGT	ACAATAATACCGAAAATAAAGT	60.0	5	0.341	216-230
51	AY486623	IAC-Hv51	(ga)17	TCAAAGATGTAAGCAGGAGTCA	AGAAGTGGCCAGCGAGAAA	65.5	7	0.698	156-170
52	AY486635	IAC-Hv52	(ca)19	AGGAGCAAACATCACAA	GCTGGAAGGGTAAACAA	57.5	6	0.389	152-168
53	AY486646	IAC-Hv53	(ca)16	CATGATGAGTGCTGAATAGAGA	TGTAAGTTTGACCAAGGATGTA	65.0	5	0.598	238-244
54	AY486666	IAC-Hv54	(ct)16	CTCTCCTCCTGCTATCCATCA	CTTTCTCCCCTTAGTCATTTCA	Not amplified			
55	AY486667	IAC-Hv55	(ga)26	AACTCCCCCATCAACCAA	GGAAATGCAAAGAAAACCAGT	64.5	6	0.651	234-244
56	AY486670	IAC-Hv56	(ga)19	TTCCAAAACACGGCTCAAT	GGCGTTCCTTTTCTCTTCC	64.5	3	0.354	250-258
57	AY486676	IAC-Hv57	(ct)15	TTTTCCTCTTCCCTTTTT	ATTCGTGTTTCTACTTTTATTT	53.5	6	0.398	244-254
58	AY486677	IAC-Hv58	(ct)18	ATTAGCTTTGCAACATTTTTCA	GGTAACGCTTCTCTTTCATTTC	63.0	4	0.512	252-246
59	AY486682	IAC-Hv59	(ct)17	TAGCAAAACAAAGCGAAAACTC	GTCCCCTGCAACTCTGTCTATC	66.0	5	0.540	200-212
60	AY486697	IAC-Hv60	(ga)20	GACCGATAATATTTCTTCACTG	AGCCATTTTCTCACCTCAT	65.0	4	0.554	176-182
61	AY486700	IAC-Hv61	(ct)17	TGGATATACCGGTAACACATTC	AACTACAGCAAAAGGCAGAGA	62.4	7	0.799	140-160
62	AY486701	IAC-Hv62	(ct)15	TCTGGCTTTGGGTCCTCTA	GGGTTTTTGGGGGTCTG	63.0	5	0.578	148-156
63	AY486702	IAC-Hv63	(ct)17	TCATCAGAAAGCAAAGAAACC	AATGCATAGGATCCACAAACT	63.5	4	0.565	208-214
64	AY486703	IAC-Hv64	(ct)23	TATGGTTTGAGCTGTGTTGTGT	TGTGCTTTTGTAATAGGGAATC	Not amplified			
65	AY486707	IAC-Hv65	(ga)16	GATGGGAAAGAGAAGAGACAAA	ATGGAAATGCGGGGGTAG	62.4	2	0.110	152-154
66	AY486708	IAC-Hv66	(ct)16	GGCGCTTGTTTTCCACTC	TTGTCTCTTCTCTTTCCCATCT	65.0	4	0.451	228-234
67	AY486720	IAC-Hv67	(ct)19	GTTGCGCAAAAATTTCTGTCTA	ACCCTTCCTGATGATTCGTGT	65.0	5	0.484	254-262
68	AY486722	IAC-Hv68	(ct)13	TGAATACTCGAGCTCTAAACTT	GGACATGATACTCGATAAACTG	65.0	4	0.338	178-184
69	AY486724	IAC-Hv69	(ct)15	TTTTACCCCTTTGGATACAGAG	GCCACAAGCTAATGATGATGAA	65.0	7	0.784	140-156
70	AY486725	IAC-Hv70	(ct)15	TCAGGGGAGATTATTTAGTTGT	GCAGGATGGAAGGGTGAG	61.8	6	0.561	290-300
71	AY486797	IAC-Hv71	(ca)22	CACTTTCGACCTCATTT	TAGTGCTTACATTTCATAGA	Not amplified			
72	AY486798	IAC-Hv72	(ca)23	CATTTCCCCTCCTCCCTCAT	TTCAGCATTTTCGTCATTTGTG	65.0	7	0.594	204-240
73	AY486802	IAC-Hv73	(ca)25	GAAGCTAACACTCAGGTAT	TCGATTAAGAAGAAGACAA	59.5	10	0.825	118-138
74	AY486807	IAC-Hv74	(ga)17	TAAAGCAGGAAAAGAAATAAAG	TCCAAGAAGACCAAGAGTT	57.6	10	0.841	170-210
75	AY486809	IAC-Hv75	(ga)18	AGAAATAGGAGCGTCAT	TGCAACAAACAGAAAGT	57.9	5	0.687	246-254
76	AY486815	IAC-Hv76	(ga)13	ACACTGACCCAACCCTTCTCT	TTCCCTTGCACTCATTCCTT	65.0	6	0.815	200-210
77	AY486816	IAC-Hv77	(ga)16	GTGCATGACAGTAATAGTTTT	CACAATGTCAATCTCAATG	Not amplified			
78	AY486819	IAC-Hv78	(ct)19	CCTCGCTTTCACTTCCCATAC	ATCCGTTGCTCTGCTGACTCTA	65.5	3	0.498	208-212
79	AY486821	IAC-Hv79	(ga)20	AGGAAAGAAAAAGCCATACC	GCAGCAGAGGACAAAACATAA	65.0	4	0.339	270-284
80	AY486822	IAC-Hv80	(ct)22	GGCTTCTACTCCTCCATCAC	AGCCCATTTCTTATTCTATTCT	62.4	10	0.610	150-172

[1]Ta - annealing temperatures, allele number.

[2]PIC - polymorphic information content

TD = Touchdown 1) 90°C, 30 s; 2) 94°C, 30 s; 60°C, 45 s (-1ºC/cycle); 72ºC for 30 s, back to step 2 for 10 x; 3) 94°C, 30 s; 50°C, 30 s; 72ºC for 45 s, back to step 3 for 25 x; 4) 62°C for 7 min; 5) 15°C soak.
