# Supplementary material for: Genetic divergence of rubber tree estimated by multivariate techniques and microsatellite markers
Source: Genet Mol Biol. 2010 Jun 1;33(2):308–18. doi: 10.1590/S1415-47572010005000039 (PMC3036869; doi:10.1590/S1415-47572010005000039)
Supplement: Figure S1 — SSR IAC-Hv72 electrophoretic profile of rubber tree genotypes on 6% polyacrylamide gel. [file gmb-33-2-308-suppl2.pdf]

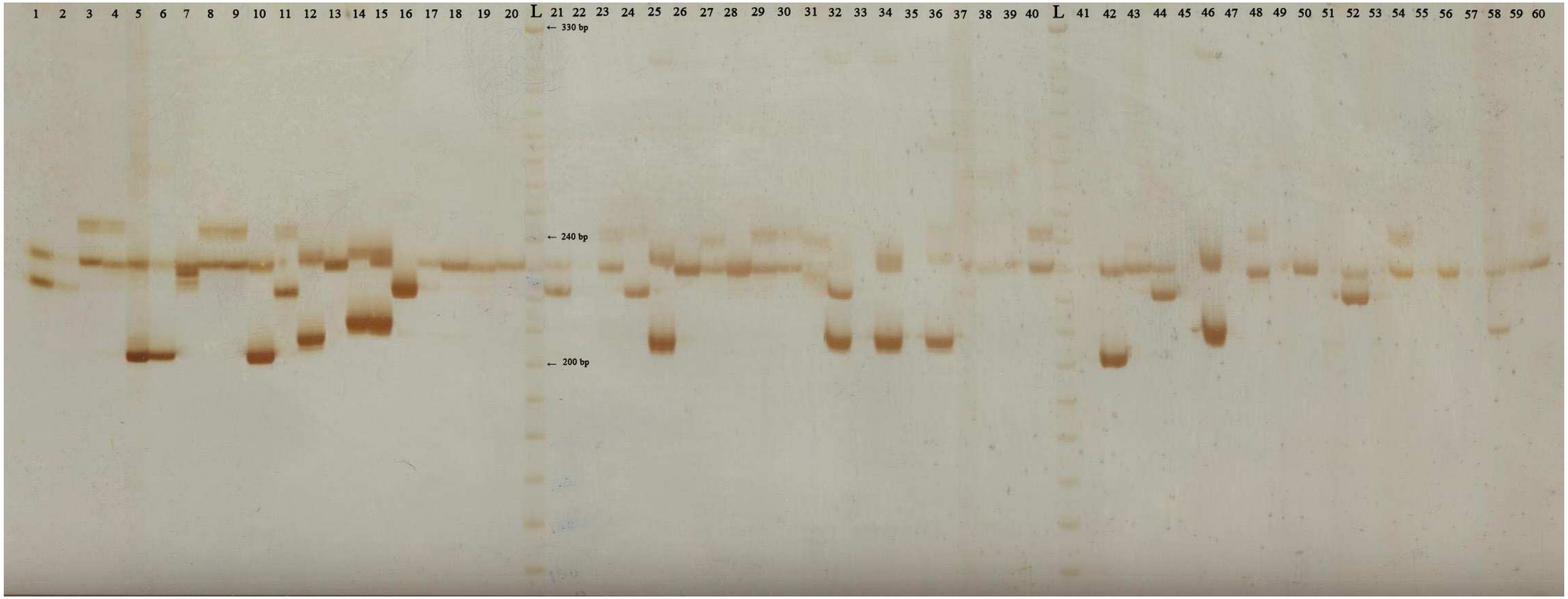

Figure S1- SSR IAC-Hv72 electrophoretic profile of rubber tree genotypes on 6% polyacrilamide gel; Lane L = 10 bp ladder marker; 1-60 = Genotype identification as referred in Table 1.
